# 100 Cases in Which Nerves Have Been Isolated by India-Rubber Sheath

**Published:** 1918-12

**Authors:** 


**100 Cases in Which Nerves Have Been Isolated by an India-rubber Sheath.** By MM. Meuriot and Platon. *Bulletin de la Société de Chirurgie de Paris.* May 14, 1918.

It is known how difficult it is for an injured nerve to recover if left in contact with bruised tissues. The usual method in such cases consists in making a new way for the operated nerve among healthy tissues. But sometimes adherences intrude between those tissues and the nerves. To avoid this, MM. Meuriot and Platon, after having operated the injured nerve, sheathe it carefully in a thin india-rubber sheet; they use sheets 20 cm. long and 5 cm. wide, which are wound around the nerve, each turn overlapping the preceding one to about one third of its length.

The tissues seemed to tolerate these sheathes remarkably well. Out of 100 cases, 93 tolerated the sheath perfectly well; only two necessitated the removal of the india-rubber.

The nerves treated in this way were : the ulnar, 32 times; the median and ulnar nerves, 13 times; the sciatic nerve, 18 times; and the plexuses twice.

This treatment had the following results :

47 cases were operated for sensory disturbances, with no further treatment than this sheathing of the nerves. In 41 cases the pains disappeared completely. In 4 cases there was no change. In 2 cases the results are unknown.

Motary disturbances occured in 74 cases; 55 were improved; 16 necessitated the suture of the nerve, and the results are not yet known.

